# If You Build It, They Will Come: Initial Experience with a Multi-Disciplinary Pediatric Neurocritical Care Follow-Up Clinic

**DOI:** 10.3390/children4090083

**Published:** 2017-09-19

**Authors:** Cydni N. Williams, Aileen Kirby, Juan Piantino

**Affiliations:** 1 Division of Pediatric Critical Care, Department of Pediatrics, Oregon Health and Science University, 707 SW Gaines St, CDRC-P, Portland, OR 97239, USA; kirbya@ohsu.edu; 2 Division of Pediatric Neurology, Department of Pediatrics, Oregon Health and Science University, 707 SW Gaines St, CDRC-P, Portland, OR 97239, USA; piantino@ohsu.edu

**Keywords:** pediatric, critical care outcomes, brain injuries, meningitis, encephalitis, stroke

## Abstract

Pediatric Neurocritical Care diagnoses account for a large proportion of intensive care admissions. Critical care survivors suffer high rates of long-term morbidity, including physical disability, cognitive impairment, and psychosocial dysfunction. To address these morbidities in Pediatric Neurocritical Care survivors, collaboration between Pediatric Neurology and Pediatric Critical Care created a multidisciplinary follow-up clinic providing specialized evaluations after discharge. Clinic referrals apply to all Pediatric Neurocritical Care patients regardless of admission severity of illness. Here, we report an initial case series, which revealed a population that is heterogenous in age, ranging from 1 month to 18 years, and in diagnoses. Traumatic brain injuries of varying severity as well as neuroinfectious and inflammatory diseases accounted for the majority of referrals. Most patients (87%) seen in the clinic had morbidities identified, requiring ongoing evaluation and expansion of the clinic. Cognitive and psychological disturbance were seen in over half of patients at the initial clinic follow-up. Sleep disturbances, daytime fatigue, headache or chronic pain, and vision or hearing concerns were also common at initial follow-up. Data from this initial population of clinic patients reiterates the need for specialized follow-up care, but also highlights the difficulties related to providing this comprehensive care and evaluating interventions to improve outcomes.

## 1. Introduction

Critical neurologic disease and injury, including infectious and inflammatory disease, traumatic brain injury (TBI), stroke, hypoxic-ischemic injury following cardiopulmonary arrest, and seizure, affect thousands of children annually and account for 20% of Pediatric Intensive Care Unit (PICU) admissions [1]. These children suffer high rates of in-hospital death and morbidity, receiving inpatient specialized Pediatric Neurocritical Care (PNCC) to optimally decrease risk of secondary brain injury and maximize recovery [1]. PICU deaths have decreased significantly over the last several decades, but have been offset by a rise in morbidities and chronic health conditions among PICU survivors, particularly with neurologic diagnoses [2,3,4]. PNCC survivors and their families suffer chronic morbidities related to physical and cognitive disability, social impairment, emotional disturbance, and psychologic disease that are collectively named Post-Intensive Care Syndrome (PICS) [5,6]. The morbidities of PICS lead to impaired quality of life and increased healthcare costs over time [7,8,9]. 

Chronic deficits have been identified in survivors of PNCC, particularly in TBI and stroke patients. Declines in functional ability are seen in a majority of severe TBI survivors [10], and new physical morbidities are seen in over one third of all PNCC survivors of TBI [11]. One third of pediatric stroke survivors have significant motor impairments one year after discharge [12]. Headaches, seizures, neurocognitive impairments, and psychosocial disturbances are also reported in TBI survivors and may persist for years [13]. Neuropsychological sequelae are also noted in stroke survivors [14]. Many of these morbidities lead to impaired quality of life [15,16]. Other conditions, such as meningitis, have more variable follow-up, making assessments of long-term outcomes more difficult, though a wide range of sequelae are reported [17,18]. 

Following PNCC, many children do not receive specialized follow-up for the long-term effects of critical illness, and research has been limited for determining effective interventions to improve outcomes. Despite receiving multidisciplinary care while an inpatient, few primary care or subspecialty providers receive training regarding PICS and most clinic environments lack the resources to identify all of these morbidities. In response to the care gap faced by PNCC survivors, we created a multidisciplinary PNCC follow-up clinic capable of assessing and addressing the multitude of morbidities faced by survivors. In this paper, we present a case series of the initial clinic experience, highlighting the heterogenous population characteristics, need for ongoing care, and future directions. 

## 2. Methods

Through collaboration between Pediatric Neurology and Pediatric Critical Care, we created a PNCC follow-up clinic, which included automatic referrals for qualified diagnoses, standardized outcome measures for longitudinal assessments, and neuropsychology services. We planned to start clinic referrals six months in advance, allowing for the creation of a half-day per month specialty clinic with our pediatric neurologist that could accommodate all referrals within two months of discharge and allow for follow-up appointments as needed. The goals of the clinic were: (1) to identify neurologic deficits and morbidities including PICS in survivors; (2) to facilitate access to therapeutic interventions for identified deficits; (3) to assist patients, families, and primary care providers with obtaining community, school, and healthcare resources. In this paper, we present a case series of PNCC patients admitted between August 2016 and January 2017 to the PICU at Doernbecher Children’s Hospital (Portland, Oregon, United States) a tertiary children’s hospital with an average of 1300 PICU admissions per year. Data presented in this paper were ascertained by a retrospective chart review of patients admitted to the PICU with a primary neurocritical care diagnosis. Morbidities reported here were ascertained from clinician documentation during the patient’s initial visit in the Pediatric Neurocritical Care follow-up clinic. This study was approved by the Oregon Health and Science University Institutional Review Board and granted waiver of informed consent (IRB No. 16173).

All PNCC survivors with TBI, neuroinfectious or inflammatory diseases, and hemorrhagic or ischemic stroke admitted to the PICU were automatically referred by a member of the PICU staff following daily census review. Survivors of status epilepticus without another established neurologist and requiring critical care for >24 h were also referred. Other patients received referrals at the discretion of attending providers. TBI is defined as a skull fracture or intracranial blood, and severity is classified based on initial hospital Glasgow Coma Scale. All patients in this study with mild TBI were diagnosed with complicated mild TBI having a skull fracture or intracranial hematoma requiring PICU admission. Patient referrals included in-person discussion with families by PICU or neurology staff regarding clinic services and potential morbidities. Referrals to the clinic were independent of inpatient Pediatric Neurology consults, but based on whether deficits were identified during hospitalization, and severity of illness. Prior to the implementation of this clinic, follow-up was variable and often based on the severity of identified symptoms during hospitalization. Some patients qualified for Pediatric Physiatry consults and inpatient rehabilitation, but many others only received follow-up through surgical subspecialties or local primary care providers. The PNCC clinic provides a standardized process to treat identified deficits and screen for subtle morbidities that arise or persist after discharge for all neurocritical care survivors. 

Neuropsychology evaluations were conducted while inpatient for children ≥3 years of age with the above diagnoses who are cognitively able to interact with the examiner. Children must be able to follow basic instructions and demonstrate basic communication skills (verbal or motor) in order to receive formal testing. Inpatient evaluations included detailed assessment of premorbid status (through parent interview or school-based assessments when available) and assessment of current deficits prior to discharge. Inpatient evaluations included counselling on identified deficits, potential morbidities, and resources for families to consult for community and school re-entry. Follow-up evaluations were completed as needed in paired clinic visits with Pediatric Neurology at intervals of four to six months. 

Outcomes in the clinic were assessed by parent and patient report, detailed examination by a pediatric neurologist, and standardized outcome measures. As no single measure can be used to screen for all morbidities associated with PICS, a combination of measures was used. Functional Status Scale (FSS) [4] scores were documented by PICU staff on admission to reflect pre-morbid status on all admissions, documented on hospital discharge by a member of the Pediatric Neurocritical Care team, and documented in clinic by a pediatric neurologist for longitudinal evaluations. Pediatric Quality of Life Inventory (PedsQL) [19] was used in clinic to screen for multi-dimensional morbidities and documented in the medical record to enable longitudinal assessments on all patients. Pediatric Glasgow Outcome Scale (GOS) scores [20] were documented in the medical record on TBI patients seen in clinic. The Standardized Concussion Assessment Tool (SCAT) was documented in the medical record on TBI patients seen in the clinic to screen for morbidities. Results for morbidities identified in clinic were compiled by a review of subjective complaints on provider documentation, objective exam findings, and review of the aforementioned standardized measures. On the PedsQL and SCAT, a positive finding was recorded for responses ≥2. 

## 3. Results

Sixty-two patients, aged 1 month to 18 years, were admitted with a qualifying diagnosis from August through January to the PICU. Forty-six (74%) patients were referred (Table 1). Seven patients died during hospitalization, seven patients were denied referral by a single insurance provider, and two were vacationing from out of state or country. The most common diagnosis was TBI. Infections included meningitis, viral encephalitis, and intracranial abscesses. Inflammatory conditions included autoimmune encephalitis and demyelinating disease. Among referrals, 31 (67%) patients completed an initial follow-up visit within two months of discharge. One patient was rescheduled outside this window, seven patients cancelled or did not attend a scheduled visit, three patients could not be contacted by a scheduler, one patient cited geographical barriers, and three patients had unknown reasons for not scheduling. Of note, 20 (65%) patients completing a clinic visit travelled >25 miles and 18 (58%) travelled >50 miles. 

During the initial referral period, 34 patients met the criteria for inpatient Neuropsychology evaluations (Table 2). Twenty-five (74%) patients completed an inpatient evaluation. One 3-year-old patient was unable to cooperate with testing, while the others were not evaluated due to the timing of discharge and neuropsychology availability. All patients missed while inpatient were mild (*n* = 7) and moderate (*n* = 2) TBI patients with short admissions, mostly over weekends when staff was not available. Two of these patients were accommodated at the initial clinic visit approximately one month after discharge. 

Four patients, all with mild TBI, were discharged from clinic after the initial visit, while the remaining 27 (87%) clinic patients had identified needs for ongoing care. Table 3 provides a summary of identified deficits and symptoms followed in these patients at the initial clinic visit 4–8 weeks after discharge. Cognitive deficits and psychological concerns were most common, occurring in over 50% of all patients seen in the clinic. Memory and attention deficits were most common among neurocognitive deficits. Anxiety and worry were also particularly common. Neurocognitive and mood disturbances are likely underrepresented in this data as these are difficult to ascertain in young children and 11 of our initial clinic patients were <3 years of age. Sleep disturbances, including increased latency, awakenings, and nightmares, were reported in over half of the patients. Daytime fatigue was also commonly reported, and did not necessarily overlap with reports of sleep disturbances. As a result of these identified morbidities, 19 (61%) patients needed referral for evaluation by other services, including physical therapy, occupational therapy, audiology, ophthalmology, otolaryngology, and counselling. Many patients (*n* = 14, 45%) required assistance with school accommodations and accessing services through school systems. Given the high prevalence of morbidities identified at the initial follow-up visit requiring ongoing care, the clinic was quickly expanded to two half-days per month to accommodate patients. Subsequently, the clinic has grown to four half-days per month with two neurology providers and will be adding a third provider from Critical Care to accommodate the increasing patient load. 

## 4. Discussion

TBI accounted for the majority of our PNCC patients. Complicated mild TBI patients seen in our PNCC clinic had high rates of deficits identified on initial follow-up evaluations, with 64% requiring ongoing clinic evaluations. These rates exceed symptom rates reported in concussion studies [21,22], and likely represents a population needing specialized follow-up potentially due to the additional morbidities of PICS. Variable follow-up of TBI patients exists nationally in the United States, ranging from primary care to surgical subspecialties. Primary care providers report discomfort caring for these patients on follow-up, as well as accessing needed resources for problems that arise in these children [23]. While concussion clinics are increasing in frequency, children with significant head injury with intracranial pathology are inherently different from the typical concussion patient, with high rates of functional impairment, psychosocial dysfunction, and neurocognitive deficits [11,13]. Concussion clinics may not have staff with experience treating patients with intracranial pathology, with PICS, or with pediatric expertise able to accommodate young patients. In our institution, patients with concussion (mild TBI without intracranial pathology) are offered follow-up through a well-established concussion clinic. Prior to PNCC clinic, many trauma patients with intracranial pathology were also referred to a concussion clinic, but due to patient complexity or young age would not receive comprehensive evaluation and would be referred to Pediatric Neurology and Neuropsychology services separately, delaying identification of impairments and access to intervention. PNCC TBI patients have variable mechanisms, severities, and locations of injuries, as well as variable developmental stages, requiring multimodal outcome assessments and arguing the importance of neurology, neuropsychology, and pediatric expertise in follow-up care. The multidisciplinary care offered in the PNCC clinic improves access to screening and intervention for this wide range of morbidities within a single clinic visit, and comprehensive assessments are performed in children of all ages. 

All of the infectious and inflammatory patients seen in clinic had morbidities identified, particularly on neuropsychology testing. Limited reports in meningitis and encephalitis patients have shown similar morbidities [9,24], but the rates of these symptoms among PNCC patients remains largely unknown. Prior to our PNCC clinic, these patients were followed by primary care physicians after discharge, as most did not receive neurology or neuropsychology consults. Stroke and hypoxic ischemic injury following cardiopulmonary arrest accounted for a smaller proportion of PNCC patients in the initial series, but survivors had important morbidities identified on follow-up. None of the initial PNCC patients with seizure qualified for referral to our clinic based on our guidelines, except one patient who died from a suspected metabolic disorder. Patients with epilepsy are known to have neurocognitive deficits, but at our institution already receive outpatient neurology and neuropsychology services, disqualifying many of our seizure admissions from referral. 

Cognitive morbidities and mood disturbances were common among PNCC patients in the initial clinic visit. These symptoms, as well as headaches and balance disturbances, are known consequences of TBI [13], but were also documented in our clinic patients with other diagnoses. Interestingly, sleep disturbances and daytime fatigue were also common. Sleep disturbances are common after TBI, though the characteristics of sleep disturbances and mechanisms of disturbances are poorly studied [25,26]. Sleep disturbances and daytime fatigue may persist for years after TBI [25], and are also associated with neurocognitive deficits and psychological disorders even among otherwise healthy children [27]. The extent to which sleep disturbances contribute to other morbidities in the PNCC population is unknown, but warrants further study. 

Systematic inpatient neuropsychology evaluations are novel in PNCC patients, but important in order to document a premorbid state for the avoidance of the “good old days bias” when seeing patients in outpatient follow-up [28]. This process identified the need for an expansion of our neuropsychology services, as many short or weekend admissions in TBI patients were unable to be accommodated due to clinician availability. However, the majority of the TBI patients that did receive evaluation had deficits while inpatient and at follow-up, distinct from the pre-morbid status documented on the initial inpatient encounter, highlighting the importance of testing these patients. 

In the future, we anticipate a further expansion of clinic access to accommodate patients with ongoing morbidities by adding neurology and critical care providers and expanding services to include physical, occupational, and speech therapy services. We are also adding neuropsychology providers to expand inpatient and outpatient access to care. The clinic was designed to assess outcomes through serial provider evaluation and longitudinal collection of standardized outcome measures, including FSS [4], PedsQL [19], GOS [20], and neuropsychological evaluations. As our patient population increases, these multi-modal outcome measures will facilitate outcomes research on both inpatient and outpatient interventions aimed at improving a range of patient outcomes to reduce chronic morbidity. 

## 5. Conclusions

The post-discharge care of PNCC survivors is variable, often leaving vulnerable patients with gaps in care. We created a PNCC follow-up clinic through collaboration between Pediatric Neurology and Pediatric Critical Care to fill this gap at our institution. Neurology and neuropsychology providers target the identification of neurologic deficits and the morbidities associated with PICS, working together to address physical, emotional, cognitive, and psychosocial concerns. Initial experience with clinic referrals reiterates the need for this specialized follow-up care, but also highlights the difficulties related to providing this comprehensive care due to the heterogenous population and multitude of morbidities identified. 

## Figures and Tables

**Table 1:** Clinic referrals and visits by diagnosis among patients meeting clinic criteria.

Diagnosis	Referred *n* = 46	Not Referred *n* = 16	Died *n* = 7	Clinic Visit Completed *n* = 31
Mild TBI	18 (39%)	4 (25%)	0	11 (35%)
Moderate TBI	4 (9%)	0	0	2 (6%)
Severe TBI	9 (20%)	0	1 (14%)	5 (16%)
Infection	6 (13%)	3 (19%)	0	5 (16%)
Inflammatory	4 (9%)	0	0	4 (13%)
Stroke	2 (4%)	0	1 (14%)	1 (3%)
Cardiopulmonary Arrest	3 (7%)	1 (6%)	4 (60%)	3 (10%)
Seizure	0	0	1 (14%)	0
Spinal Cord Injury	0	1 (6%)	0	0

TBI: Traumatic Brain Injury.

**Table 2:** Inpatient neuropsychology evaluation by diagnosis.

Diagnosis	Inpatient Evaluation Qualified ^a^ *n* = 34	Evaluation Completed *n* = 25
Mild TBI	12 (35%)	5 (20%)
Moderate TBI	4 (12%)	2 (8%)
Severe TBI	5 (15%)	5 (20%)
Infection	5 (15%)	5 (20%)
Inflammatory	3 (9%)	3 (12%)
Stroke	2 (6%)	2 (8%)
Cardiopulmonary Arrest	2 (6%)	2 (8%)
Spinal Cord Injury	1 (3%)	1 (4%)

^a^: Qualified for neuropsychology evaluation if ≥3 years of age and able to interact with examiner.

**Table 3:** Morbidities identified among 31 clinic patients.

Neurocognitive Deficits	
Attention	10 (32%)
Memory	9 (29%)
Other	5 (16%)
Mood Disturbance	
Anxiety	11 (35%)
Behavior	8 (26%)
Sadness/Depression	5 (16%)
Sleep Disturbance	16 (52%)
Vision or Hearing	10 (32%)
Daytime Fatigue	9 (29%)
Headache or Chronic Pain	7 (23%)
Balance Disturbance	6 (19%)
Developmental Concerns	5 (16%)
Seizure management	4 (13%)
Weakness	3 (10%)
